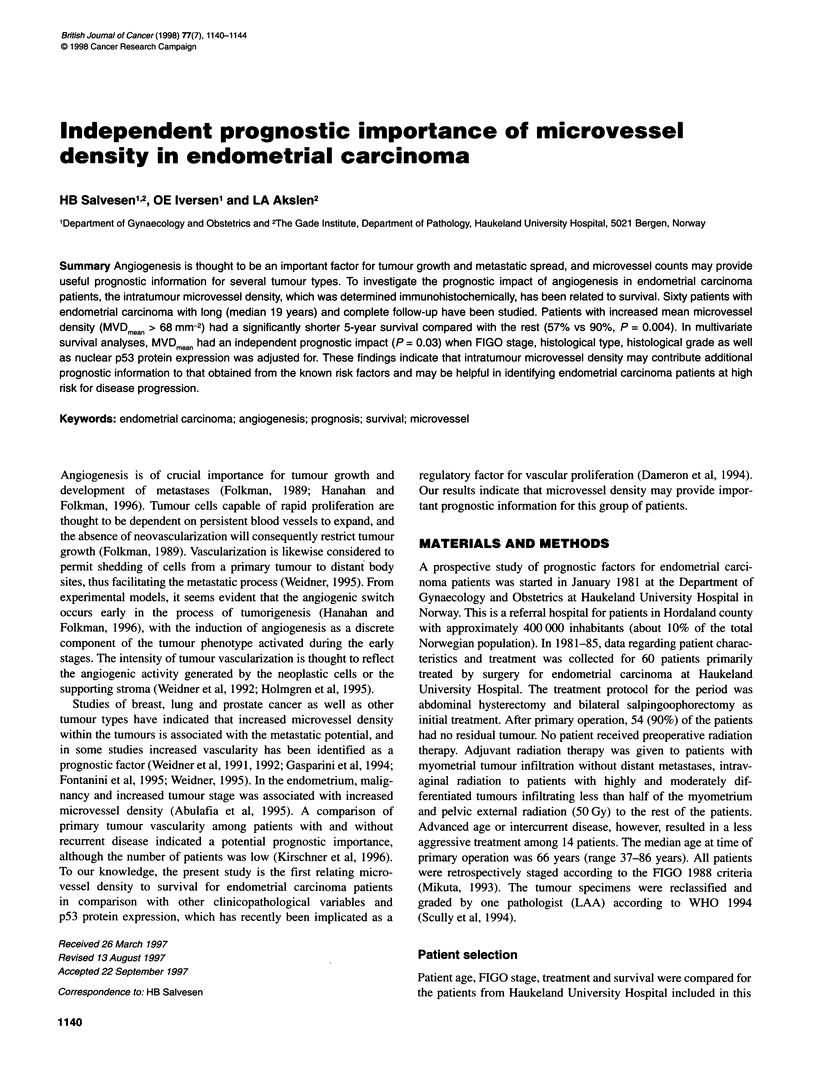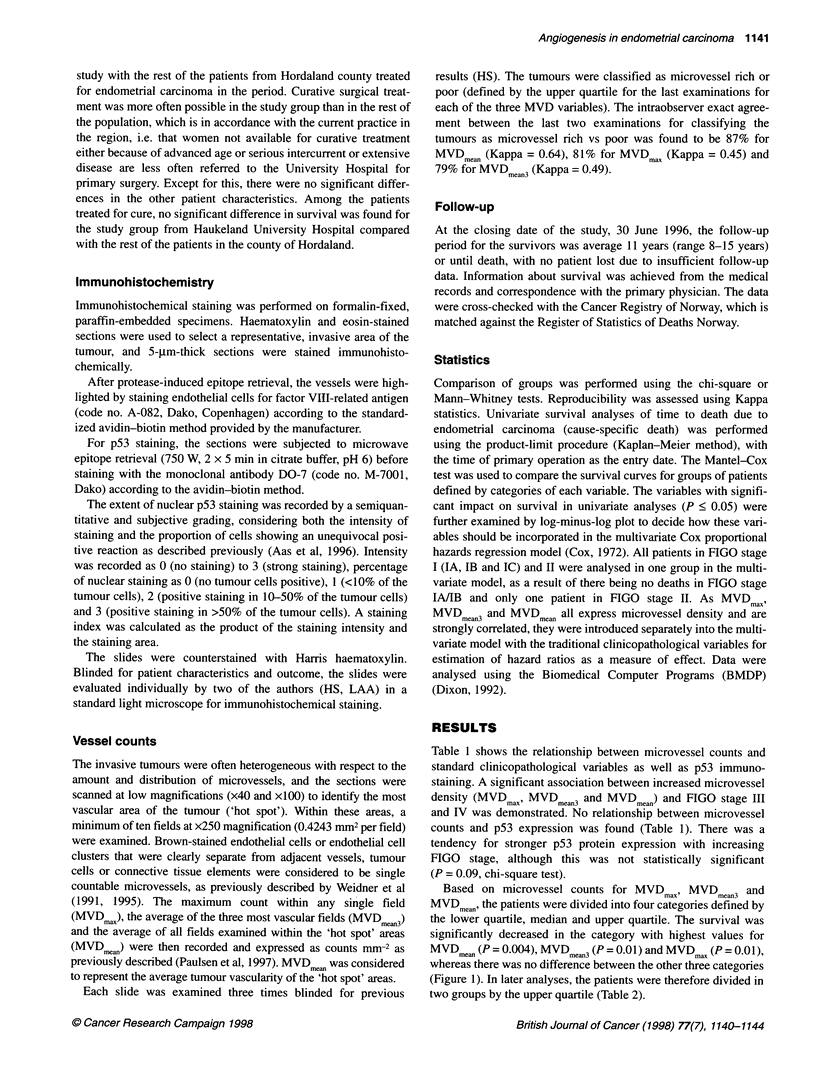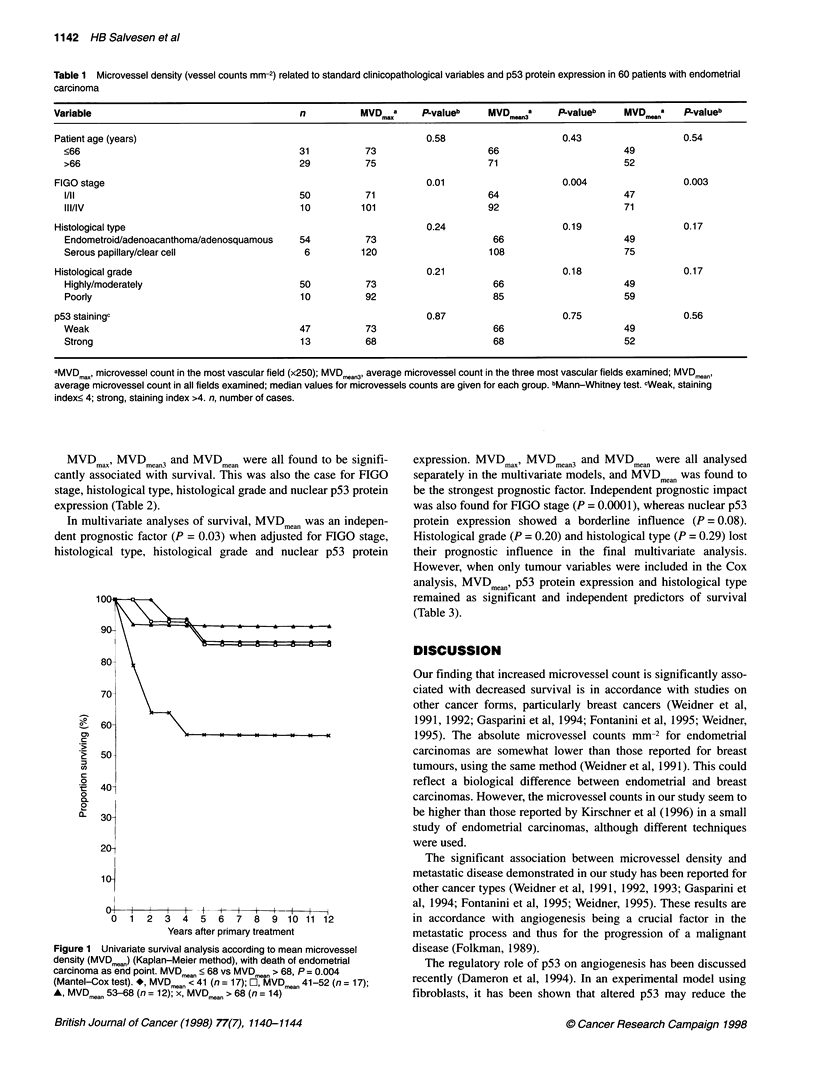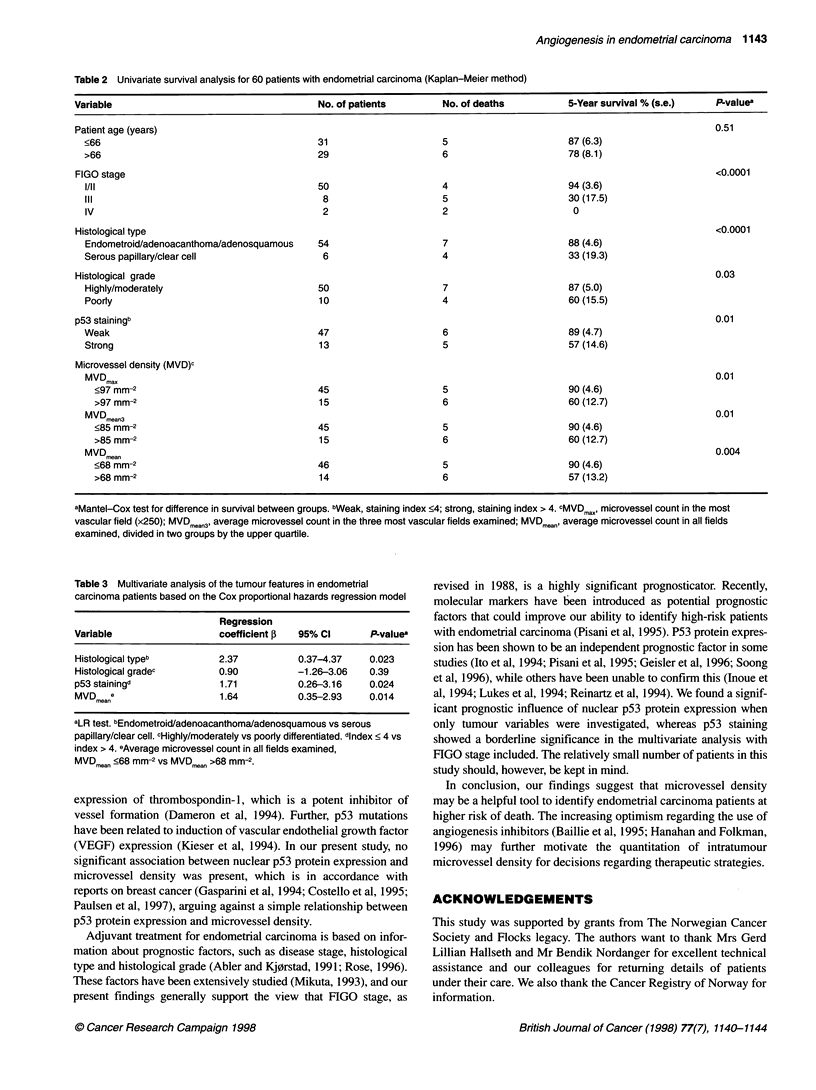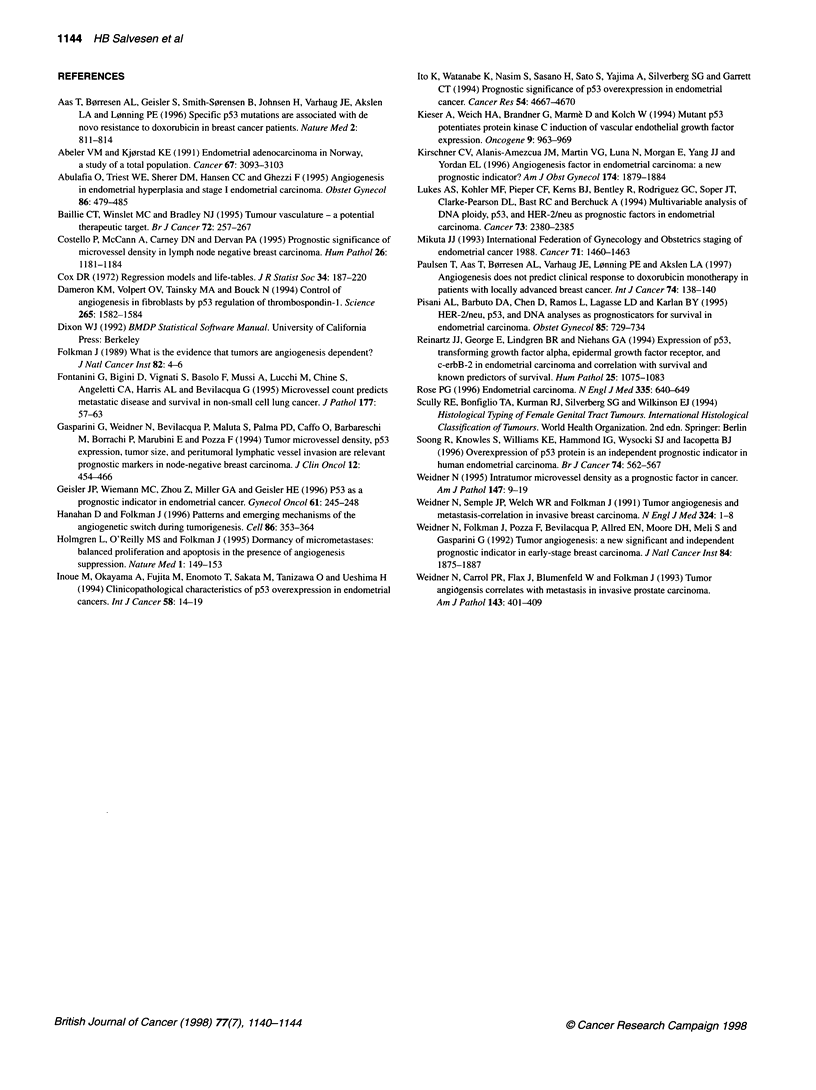# Independent prognostic importance of microvessel density in endometrial carcinoma.

**DOI:** 10.1038/bjc.1998.189

**Published:** 1998-04

**Authors:** H. B. Salvesen, O. E. Iversen, L. A. Akslen

**Affiliations:** Department of Gynaecology and Obstetrics, The Gade Institute, Haukeland University Hospital, Bergen, Norway.

## Abstract

Angiogenesis is thought to be an important factor for tumour growth and metastatic spread, and microvessel counts may provide useful prognostic information for several tumour types. To investigate the prognostic impact of angiogenesis in endometrial carcinoma patients, the intratumour microvessel density, which was determined immunohistochemically, has been related to survival. Sixty patients with endometrial carcinoma with long (median 19 years) and complete follow-up have been studied. Patients with increased mean microvessel density (MVDmean > 68 mm2) had a significantly shorter 5-year survival compared with the rest (57% vs 90%, P = 0.004). In multivariate survival analyses, MVDmean had an independent prognostic impact (P = 0.03) when FIGO stage, histological type, histological grade as well as nuclear p53 protein expression was adjusted for. These findings indicate that intratumour microvessel density may contribute additional prognostic information to that obtained from the known risk factors and may be helpful in identifying endometrial carcinoma patients at high risk for disease progression.


British Journal of Cancer (1998) 77(7), 1140–1144
© 1998 Cancer Research Campaign

### Independent prognostic importance of microvessel density in endometrial carcinoma

**HB Salvesen[1,2], OE Iversen[1] and LA Akslen[2]**

[1]Department of Gynaecology and Obstetrics and [2]The Gade Institute, Department of Pathology, Haukeland University Hospital, 5021 Bergen, Norway

**Summary** Angiogenesis is thought to be an important factor for tumour growth and metastatic spread, and microvessel counts may provide useful prognostic information for several tumour types. To investigate the prognostic impact of angiogenesis in endometrial carcinoma patients, the intratumour microvessel density, which was determined immunohistochemically, has been related to survival. Sixty patients with endometrial carcinoma with long (median 19 years) and complete follow-up have been studied. Patients with increased mean microvessel density ($MVD_{mean}$ > 68 $mm^{-2}$) had a significantly shorter 5-year survival compared with the rest (57% vs 90%, $P$ = 0.004). In multivariate survival analyses, $MVD_{mean}$ had an independent prognostic impact ($P$ = 0.03) when FIGO stage, histological type, histological grade as well as nuclear p53 protein expression was adjusted for. These findings indicate that intratumour microvessel density may contribute additional prognostic information to that obtained from the known risk factors and may be helpful in identifying endometrial carcinoma patients at high risk for disease progression.

**Keywords:** endometrial carcinoma; angiogenesis; prognosis; survival; microvessel

Angiogenesis is of crucial importance for tumour growth and development of metastases (Folkman, 1989; Hanahan and Folkman, 1996). Tumour cells capable of rapid proliferation are thought to be dependent on persistent blood vessels to expand, and the absence of neovascularization will consequently restrict tumour growth (Folkman, 1989). Vascularization is likewise considered to permit shedding of cells from a primary tumour to distant body sites, thus facilitating the metastatic process (Weidner, 1995). From experimental models, it seems evident that the angiogenic switch occurs early in the process of tumorigenesis (Hanahan and Folkman, 1996), with the induction of angiogenesis as a discrete component of the tumour phenotype activated during the early stages. The intensity of tumour vascularization is thought to reflect the angiogenic activity generated by the neoplastic cells or the supporting stroma (Weidner et al, 1992; Holmgren et al, 1995).

Studies of breast, lung and prostate cancer as well as other tumour types have indicated that increased microvessel density within the tumours is associated with the metastatic potential, and in some studies increased vascularity has been identified as a prognostic factor (Weidner et al, 1991, 1992; Gasparini et al, 1994; Fontanini et al, 1995; Weidner, 1995). In the endometrium, malignancy and increased tumour stage was associated with increased microvessel density (Abulafia et al, 1995). A comparison of primary tumour vascularity among patients with and without recurrent disease indicated a potential prognostic importance, although the number of patients was low (Kirschner et al, 1996). To our knowledge, the present study is the first relating microvessel density to survival for endometrial carcinoma patients in comparison with other clinicopathological variables and p53 protein expression, which has recently been implicated as a regulatory factor for vascular proliferation (Dameron et al, 1994). Our results indicate that microvessel density may provide important prognostic information for this group of patients.

*Received 26 March 1997*
*Revised 13 August 1997*
*Accepted 22 September 1997*

*Correspondence to:* HB Salvesen

### MATERIALS AND METHODS

A prospective study of prognostic factors for endometrial carcinoma patients was started in January 1981 at the Department of Gynaecology and Obstetrics at Haukeland University Hospital in Norway. This is a referral hospital for patients in Hordaland county with approximately 400 000 inhabitants (about 10% of the total Norwegian population). In 1981–85, data regarding patient characteristics and treatment was collected for 60 patients primarily treated by surgery for endometrial carcinoma at Haukeland University Hospital. The treatment protocol for the period was abdominal hysterectomy and bilateral salpingoophorectomy as initial treatment. After primary operation, 54 (90%) of the patients had no residual tumour. No patient received preoperative radiation therapy. Adjuvant radiation therapy was given to patients with myometrial tumour infiltration without distant metastases, intravaginal radiation to patients with highly and moderately differentiated tumours infiltrating less than half of the myometrium and pelvic external radiation (50 Gy) to the rest of the patients. Advanced age or intercurrent disease, however, resulted in a less aggressive treatment among 14 patients. The median age at time of primary operation was 66 years (range 37–86 years). All patients were retrospectively staged according to the FIGO 1988 criteria (Mikuta, 1993). The tumour specimens were reclassified and graded by one pathologist (LAA) according to WHO 1994 (Scully et al, 1994).

### Patient selection

Patient age, FIGO stage, treatment and survival were compared for the patients from Haukeland University Hospital included in this study with the rest of the patients from Hordaland county treated for endometrial carcinoma in the period. Curative surgical treatment was more often possible in the study group than in the rest of the population, which is in accordance with the current practice in the region, i.e. that women not available for curative treatment either because of advanced age or serious intercurrent or extensive disease are less often referred to the University Hospital for primary surgery. Except for this, there were no significant differences in the other patient characteristics. Among the patients treated for cure, no significant difference in survival was found for the study group from Haukeland University Hospital compared with the rest of the patients in the county of Hordaland.

### Immunohistochemistry

Immunohistochemical staining was performed on formalin-fixed, paraffin-embedded specimens. Haematoxylin and eosin-stained sections were used to select a representative, invasive area of the tumour, and 5-μm-thick sections were stained immunohistochemically.

After protease-induced epitope retrieval, the vessels were highlighted by staining endothelial cells for factor VIII-related antigen (code no. A-082, Dako, Copenhagen) according to the standardized avidin–biotin method provided by the manufacturer.

For p53 staining, the sections were subjected to microwave epitope retrieval (750 W, 2 × 5 min in citrate buffer, pH 6) before staining with the monoclonal antibody DO-7 (code no. M-7001, Dako) according to the avidin–biotin method.

The extent of nuclear p53 staining was recorded by a semiquantitative and subjective grading, considering both the intensity of staining and the proportion of cells showing an unequivocal positive reaction as described previously (Aas et al, 1996). Intensity was recorded as 0 (no staining) to 3 (strong staining), percentage of nuclear staining as 0 (no tumour cells positive), 1 (<10% of the tumour cells), 2 (positive staining in 10–50% of the tumour cells) and 3 (positive staining in >50% of the tumour cells). A staining index was calculated as the product of the staining intensity and the staining area.

The slides were counterstained with Harris haematoxylin. Blinded for patient characteristics and outcome, the slides were evaluated individually by two of the authors (HS, LAA) in a standard light microscope for immunohistochemical staining.

### Vessel counts

The invasive tumours were often heterogeneous with respect to the amount and distribution of microvessels, and the sections were scanned at low magnifications (×40 and ×100) to identify the most vascular area of the tumour ('hot spot'). Within these areas, a minimum of ten fields at ×250 magnification (0.4243 $mm^2$ per field) were examined. Brown-stained endothelial cells or endothelial cell clusters that were clearly separate from adjacent vessels, tumour cells or connective tissue elements were considered to be single countable microvessels, as previously described by Weidner et al (1991, 1995). The maximum count within any single field ($MVD_{max}$), the average of the three most vascular fields ($MVD_{mean3}$) and the average of all fields examined within the 'hot spot' areas ($MVD_{mean}$) were then recorded and expressed as counts $mm^{-2}$ as previously described (Paulsen et al, 1997). $MVD_{mean}$ was considered to represent the average tumour vascularity of the 'hot spot' areas.

Each slide was examined three times blinded for previous results (HS). The tumours were classified as microvessel rich or poor (defined by the upper quartile for the last examinations for each of the three MVD variables). The intraobserver exact agreement between the last two examinations for classifying the tumours as microvessel rich vs poor was found to be 87% for $MVD_{mean}$ (Kappa = 0.64), 81% for $MVD_{max}$ (Kappa = 0.45) and 79% for $MVD_{mean3}$ (Kappa = 0.49).

### Follow-up

At the closing date of the study, 30 June 1996, the follow-up period for the survivors was average 11 years (range 8–15 years) or until death, with no patient lost due to insufficient follow-up data. Information about survival was achieved from the medical records and correspondence with the primary physician. The data were cross-checked with the Cancer Registry of Norway, which is matched against the Register of Statistics of Deaths Norway.

### Statistics

Comparison of groups was performed using the chi-square or Mann–Whitney tests. Reproducibility was assessed using Kappa statistics. Univariate survival analyses of time to death due to endometrial carcinoma (cause-specific death) was performed using the product-limit procedure (Kaplan–Meier method), with the time of primary operation as the entry date. The Mantel–Cox test was used to compare the survival curves for groups of patients defined by categories of each variable. The variables with significant impact on survival in univariate analyses ($P \leq 0.05$) were further examined by log-minus-log plot to decide how these variables should be incorporated in the multivariate Cox proportional hazards regression model (Cox, 1972). All patients in FIGO stage I (IA, IB and IC) and II were analysed in one group in the multivariate model, as a result of there being no deaths in FIGO stage IA/IB and only one patient in FIGO stage II. As $MVD_{max}$, $MVD_{mean3}$ and $MVD_{mean}$ all express microvessel density and are strongly correlated, they were introduced separately into the multivariate model with the traditional clinicopathological variables for estimation of hazard ratios as a measure of effect. Data were analysed using the Biomedical Computer Programs (BMDP) (Dixon, 1992).

### RESULTS

Table 1 shows the relationship between microvessel counts and standard clinicopathological variables as well as p53 immunostaining. A significant association between increased microvessel density ($MVD_{max}$, $MVD_{mean3}$ and $MVD_{mean}$) and FIGO stage III and IV was demonstrated. No relationship between microvessel counts and p53 expression was found (Table 1). There was a tendency for stronger p53 protein expression with increasing FIGO stage, although this was not statistically significant ($P = 0.09$, chi-square test).

Based on microvessel counts for $MVD_{max}$, $MVD_{mean3}$ and $MVD_{mean}$, the patients were divided into four categories defined by the lower quartile, median and upper quartile. The survival was significantly decreased in the category with highest values for $MVD_{mean}$ ($P = 0.004$), $MVD_{mean3}$ ($P = 0.01$) and $MVD_{max}$ ($P = 0.01$), whereas there was no difference between the other three categories (Figure 1). In later analyses, the patients were therefore divided in two groups by the upper quartile (Table 2).

© Cancer Research Campaign 1998

**Table 1** Microvessel density (vessel counts $mm^{-2}$) related to standard clinicopathological variables and p53 protein expression in 60 patients with endometrial carcinoma

| Variable | n | $MVD_{max}$[a] | P-value[b] | $MVD_{mean3}$[a] | P-value[b] | $MVD_{mean}$[a] | P-value[b] |
|---|---|---|---|---|---|---|---|
| Patient age (years) | | | 0.58 | | 0.43 | | 0.54 |
| ≤66 | 31 | 73 | | 66 | | 49 | |
| >66 | 29 | 75 | | 71 | | 52 | |
| FIGO stage | | | 0.01 | | 0.004 | | 0.003 |
| I/II | 50 | 71 | | 64 | | 47 | |
| III/IV | 10 | 101 | | 92 | | 71 | |
| Histological type | | | 0.24 | | 0.19 | | 0.17 |
| Endometroid/adenoacanthoma/adenosquamous | 54 | 73 | | 66 | | 49 | |
| Serous papillary/clear cell | 6 | 120 | | 108 | | 75 | |
| Histological grade | | | 0.21 | | 0.18 | | 0.17 |
| Highly/moderately | 50 | 73 | | 66 | | 49 | |
| Poorly | 10 | 92 | | 85 | | 59 | |
| p53 staining[c] | | | 0.87 | | 0.75 | | 0.56 |
| Weak | 47 | 73 | | 66 | | 49 | |
| Strong | 13 | 68 | | 68 | | 52 | |

[a]$MVD_{max}$, microvessel count in the most vascular field (×250); $MVD_{mean3}$, average microvessel count in the three most vascular fields examined; $MVD_{mean}$, average microvessel count in all fields examined; median values for microvessels counts are given for each group. [b]Mann–Whitney test. [c]Weak, staining index≤ 4; strong, staining index >4. *n*, number of cases.

$MVD_{max}$, $MVD_{mean3}$ and $MVD_{mean}$ were all found to be significantly associated with survival. This was also the case for FIGO stage, histological type, histological grade and nuclear p53 protein expression (Table 2).

In multivariate analyses of survival, $MVD_{mean}$ was an independent prognostic factor ($P$ = 0.03) when adjusted for FIGO stage, histological type, histological grade and nuclear p53 protein expression. $MVD_{max}$, $MVD_{mean3}$ and $MVD_{mean}$ were all analysed separately in the multivariate models, and $MVD_{mean}$ was found to be the strongest prognostic factor. Independent prognostic impact was also found for FIGO stage ($P$ = 0.0001), whereas nuclear p53 protein expression showed a borderline influence ($P$ = 0.08). Histological grade ($P$ = 0.20) and histological type ($P$ = 0.29) lost their prognostic influence in the final multivariate analysis. However, when only tumour variables were included in the Cox analysis, $MVD_{mean}$, p53 protein expression and histological type remained as significant and independent predictors of survival (Table 3).

**Figure 1** Univariate survival analysis according to mean microvessel density ($MVD_{mean}$) (Kaplan–Meier method), with death of endometrial carcinoma as end point. $MVD_{mean}$ ≤ 68 vs $MVD_{mean}$ > 68, $P$ = 0.004 (Mantel–Cox test). ◆, $MVD_{mean}$ < 41 ($n$ = 17); □, $MVD_{mean}$ 41–52 ($n$ = 17); ▲, $MVD_{mean}$ 53–68 ($n$ = 12); ×, $MVD_{mean}$ > 68 ($n$ = 14)

### DISCUSSION

Our finding that increased microvessel count is significantly associated with decreased survival is in accordance with studies on other cancer forms, particularly breast cancers (Weidner et al, 1991, 1992; Gasparini et al, 1994; Fontanini et al, 1995; Weidner, 1995). The absolute microvessel counts $mm^{-2}$ for endometrial carcinomas are somewhat lower than those reported for breast tumours, using the same method (Weidner et al, 1991). This could reflect a biological difference between endometrial and breast carcinomas. However, the microvessel counts in our study seem to be higher than those reported by Kirschner et al (1996) in a small study of endometrial carcinomas, although different techniques were used.

The significant association between microvessel density and metastatic disease demonstrated in our study has been reported for other cancer types (Weidner et al, 1991, 1992, 1993; Gasparini et al, 1994; Fontanini et al, 1995; Weidner, 1995). These results are in accordance with angiogenesis being a crucial factor in the metastatic process and thus for the progression of a malignant disease (Folkman, 1989).

The regulatory role of p53 on angiogenesis has been discussed recently (Dameron et al, 1994). In an experimental model using fibroblasts, it has been shown that altered p53 may reduce the

© Cancer Research Campaign 1998

**Table 2** Univariate survival analysis for 60 patients with endometrial carcinoma (Kaplan–Meier method)

| Variable | No. of patients | No. of deaths | 5-Year survival % (s.e.) | *P*-value[a] |
|---|---|---|---|---|
| Patient age (years) | | | | 0.51 |
| ≤66 | 31 | 5 | 87 (6.3) | |
| >66 | 29 | 6 | 78 (8.1) | |
| FIGO stage | | | | <0.0001 |
| I/II | 50 | 4 | 94 (3.6) | |
| III | 8 | 5 | 30 (17.5) | |
| IV | 2 | 2 | 0 | |
| Histological type | | | | <0.0001 |
| Endometroid/adenoacanthoma/adenosquamous | 54 | 7 | 88 (4.6) | |
| Serous papillary/clear cell | 6 | 4 | 33 (19.3) | |
| Histological grade | | | | 0.03 |
| Highly/moderately | 50 | 7 | 87 (5.0) | |
| Poorly | 10 | 4 | 60 (15.5) | |
| p53 staining[b] | | | | 0.01 |
| Weak | 47 | 6 | 89 (4.7) | |
| Strong | 13 | 5 | 57 (14.6) | |
| Microvessel density (MVD)[c] | | | | |
| $MVD_{max}$ | | | | 0.01 |
| ≤97 $mm^{-2}$ | 45 | 5 | 90 (4.6) | |
| >97 $mm^{-2}$ | 15 | 6 | 60 (12.7) | |
| $MVD_{mean3}$ | | | | 0.01 |
| ≤85 $mm^{-2}$ | 45 | 5 | 90 (4.6) | |
| >85 $mm^{-2}$ | 15 | 6 | 60 (12.7) | |
| $MVD_{mean}$ | | | | 0.004 |
| ≤68 $mm^{-2}$ | 46 | 5 | 90 (4.6) | |
| >68 $mm^{-2}$ | 14 | 6 | 57 (13.2) | |

[a]Mantel–Cox test for difference in survival between groups. [b]Weak, staining index ≤4; strong, staining index > 4. [c]$MVD_{max}$, microvessel count in the most vascular field (×250); $MVD_{mean3}$, average microvessel count in the three most vascular fields examined; $MVD_{mean}$, average microvessel count in all fields examined, divided in two groups by the upper quartile.

**Table 3** Multivariate analysis of the tumour features in endometrial carcinoma patients based on the Cox proportional hazards regression model

| Variable | Regression coefficient β | 95% CI | *P*-value[a] |
|---|---|---|---|
| Histological type[b] | 2.37 | 0.37–4.37 | 0.023 |
| Histological grade[c] | 0.90 | −1.26–3.06 | 0.39 |
| p53 staining[d] | 1.71 | 0.26–3.16 | 0.024 |
| $MVD_{mean}$[e] | 1.64 | 0.35–2.93 | 0.014 |

[a]LR test. [b]Endometroid/adenoacanthoma/adenosquamous vs serous papillary/clear cell. [c]Highly/moderately vs poorly differentiated. [d]Index ≤ 4 vs index > 4. [e]Average microvessel count in all fields examined, $MVD_{mean}$ ≤68 $mm^{-2}$ vs $MVD_{mean}$ >68 $mm^{-2}$.

expression of thrombospondin-1, which is a potent inhibitor of vessel formation (Dameron et al, 1994). Further, p53 mutations have been related to induction of vascular endothelial growth factor (VEGF) expression (Kieser et al, 1994). In our present study, no significant association between nuclear p53 protein expression and microvessel density was present, which is in accordance with reports on breast cancer (Gasparini et al, 1994; Costello et al, 1995; Paulsen et al, 1997), arguing against a simple relationship between p53 protein expression and microvessel density.

Adjuvant treatment for endometrial carcinoma is based on information about prognostic factors, such as disease stage, histological type and histological grade (Abler and Kjørstad, 1991; Rose, 1996). These factors have been extensively studied (Mikuta, 1993), and our present findings generally support the view that FIGO stage, as revised in 1988, is a highly significant prognosticator. Recently, molecular markers have been introduced as potential prognostic factors that could improve our ability to identify high-risk patients with endometrial carcinoma (Pisani et al, 1995). P53 protein expression has been shown to be an independent prognostic factor in some studies (Ito et al, 1994; Pisani et al, 1995; Geisler et al, 1996; Soong et al, 1996), while others have been unable to confirm this (Inoue et al, 1994; Lukes et al, 1994; Reinartz et al, 1994). We found a significant prognostic influence of nuclear p53 protein expression when only tumour variables were investigated, whereas p53 staining showed a borderline significance in the multivariate analysis with FIGO stage included. The relatively small number of patients in this study should, however, be kept in mind.

In conclusion, our findings suggest that microvessel density may be a helpful tool to identify endometrial carcinoma patients at higher risk of death. The increasing optimism regarding the use of angiogenesis inhibitors (Baillie et al, 1995; Hanahan and Folkman, 1996) may further motivate the quantitation of intratumour microvessel density for decisions regarding therapeutic strategies.

### ACKNOWLEDGEMENTS

This study was supported by grants from The Norwegian Cancer Society and Flocks legacy. The authors want to thank Mrs Gerd Lillian Hallseth and Mr Bendik Nordanger for excellent technical assistance and our colleagues for returning details of patients under their care. We also thank the Cancer Registry of Norway for information.

*© Cancer Research Campaign 1998*

### REFERENCES

Aas T, Børresen AL, Geisler S, Smith-Sørensen B, Johnsen H, Varhaug JE, Akslen LA and Lønning PE (1996) Specific p53 mutations are associated with de novo resistance to doxorubicin in breast cancer patients. *Nature Med* **2**: 811–814

Abeler VM and Kjørstad KE (1991) Endometrial adenocarcinoma in Norway, a study of a total population. *Cancer* **67**: 3093–3103

Abulafia O, Triest WE, Sherer DM, Hansen CC and Ghezzi F (1995) Angiogenesis in endometrial hyperplasia and stage I endometrial carcinoma. *Obstet Gynecol* **86**: 479–485

Baillie CT, Winslet MC and Bradley NJ (1995) Tumour vasculature – a potential therapeutic target. *Br J Cancer* **72**: 257–267

Costello P, McCann A, Carney DN and Dervan PA (1995) Prognostic significance of microvessel density in lymph node negative breast carcinoma. *Hum Pathol* **26**: 1181–1184

Cox DR (1972) Regression models and life-tables. *J R Statist Soc* **34**: 187–220

Dameron KM, Volpert OV, Tainsky MA and Bouck N (1994) Control of angiogenesis in fibroblasts by p53 regulation of thrombospondin-1. *Science* **265**: 1582–1584

Dixon WJ (1992) *BMDP Statistical Software Manual*. University of California Press: Berkeley

Folkman J (1989) What is the evidence that tumors are angiogenesis dependent? *J Natl Cancer Inst* **82**: 4–6

Fontanini G, Bigini D, Vignati S, Basolo F, Mussi A, Lucchi M, Chine S, Angeletti CA, Harris AL and Bevilacqua G (1995) Microvessel count predicts metastatic disease and survival in non-small cell lung cancer. *J Pathol* **177**: 57–63

Gasparini G, Weidner N, Bevilacqua P, Maluta S, Palma PD, Caffo O, Barbareschi M, Borrachi P, Marubini E and Pozza F (1994) Tumor microvessel density, p53 expression, tumor size, and peritumoral lymphatic vessel invasion are relevant prognostic markers in node-negative breast carcinoma. *J Clin Oncol* **12**: 454–466

Geisler JP, Wiemann MC, Zhou Z, Miller GA and Geisler HE (1996) P53 as a prognostic indicator in endometrial cancer. *Gynecol Oncol* **61**: 245–248

Hanahan D and Folkman J (1996) Patterns and emerging mechanisms of the angiogenetic switch during tumorigenesis. *Cell* **86**: 353–364

Holmgren L, O'Reilly MS and Folkman J (1995) Dormancy of micrometastases: balanced proliferation and apoptosis in the presence of angiogenesis suppression. *Nature Med* **1**: 149–153

Inoue M, Okayama A, Fujita M, Enomoto T, Sakata M, Tanizawa O and Ueshima H (1994) Clinicopathological characteristics of p53 overexpression in endometrial cancers. *Int J Cancer* **58**: 14–19

Ito K, Watanabe K, Nasim S, Sasano H, Sato S, Yajima A, Silverberg SG and Garrett CT (1994) Prognostic significance of p53 overexpression in endometrial cancer. *Cancer Res* **54**: 4667–4670

Kieser A, Weich HA, Brandner G, Marmè D and Kolch W (1994) Mutant p53 potentiates protein kinase C induction of vascular endothelial growth factor expression. *Oncogene* **9**: 963–969

Kirschner CV, Alanis-Amezcua JM, Martin VG, Luna N, Morgan E, Yang JJ and Yordan EL (1996) Angiogenesis factor in endometrial carcinoma: a new prognostic indicator? *Am J Obst Gynecol* **174**: 1879–1884

Lukes AS, Kohler MF, Pieper CF, Kerns BJ, Bentley R, Rodriguez GC, Soper JT, Clarke-Pearson DL, Bast RC and Berchuck A (1994) Multivariable analysis of DNA ploidy, p53, and HER-2/neu as prognostic factors in endometrial carcinoma. *Cancer* **73**: 2380–2385

Mikuta JJ (1993) International Federation of Gynecology and Obstetrics staging of endometrial cancer 1988. *Cancer* **71**: 1460–1463

Paulsen T, Aas T, Børresen AL, Varhaug JE, Lønning PE and Akslen LA (1997) Angiogenesis does not predict clinical response to doxorubicin monotherapy in patients with locally advanced breast cancer. *Int J Cancer* **74**: 138–140

Pisani AL, Barbuto DA, Chen D, Ramos L, Lagasse LD and Karlan BY (1995) HER-2/neu, p53, and DNA analyses as prognosticators for survival in endometrial carcinoma. *Obstet Gynecol* **85**: 729–734

Reinartz JJ, George E, Lindgren BR and Niehans GA (1994) Expression of p53, transforming growth factor alpha, epidermal growth factor receptor, and c-erbB-2 in endometrial carcinoma and correlation with survival and known predictors of survival. *Hum Pathol* **25**: 1075–1083

Rose PG (1996) Endometrial carcinoma. *N Engl J Med* **335**: 640–649

Scully RE, Bonfiglio TA, Kurman RJ, Silverberg SG and Wilkinson EJ (1994) *Histological Typing of Female Genital Tract Tumours. International Histological Classification of Tumours*. World Health Organization. 2nd edn. Springer: Berlin

Soong R, Knowles S, Williams KE, Hammond IG, Wysocki SJ and Iacopetta BJ (1996) Overexpression of p53 protein is an independent prognostic indicator in human endometrial carcinoma. *Br J Cancer* **74**: 562–567

Weidner N (1995) Intratumor microvessel density as a prognostic factor in cancer. *Am J Pathol* **147**: 9–19

Weidner N, Semple JP, Welch WR and Folkman J (1991) Tumor angiogenesis and metastasis-correlation in invasive breast carcinoma. *N Engl J Med* **324**: 1–8

Weidner N, Folkman J, Pozza F, Bevilacqua P, Allred EN, Moore DH, Meli S and Gasparini G (1992) Tumor angiogenesis: a new significant and independent prognostic indicator in early-stage breast carcinoma. *J Natl Cancer Inst* **84**: 1875–1887

Weidner N, Carrol PR, Flax J, Blumenfeld W and Folkman J (1993) Tumor angiögensis correlates with metastasis in invasive prostate carcinoma. *Am J Pathol* **143**: 401–409

© Cancer Research Campaign 1998