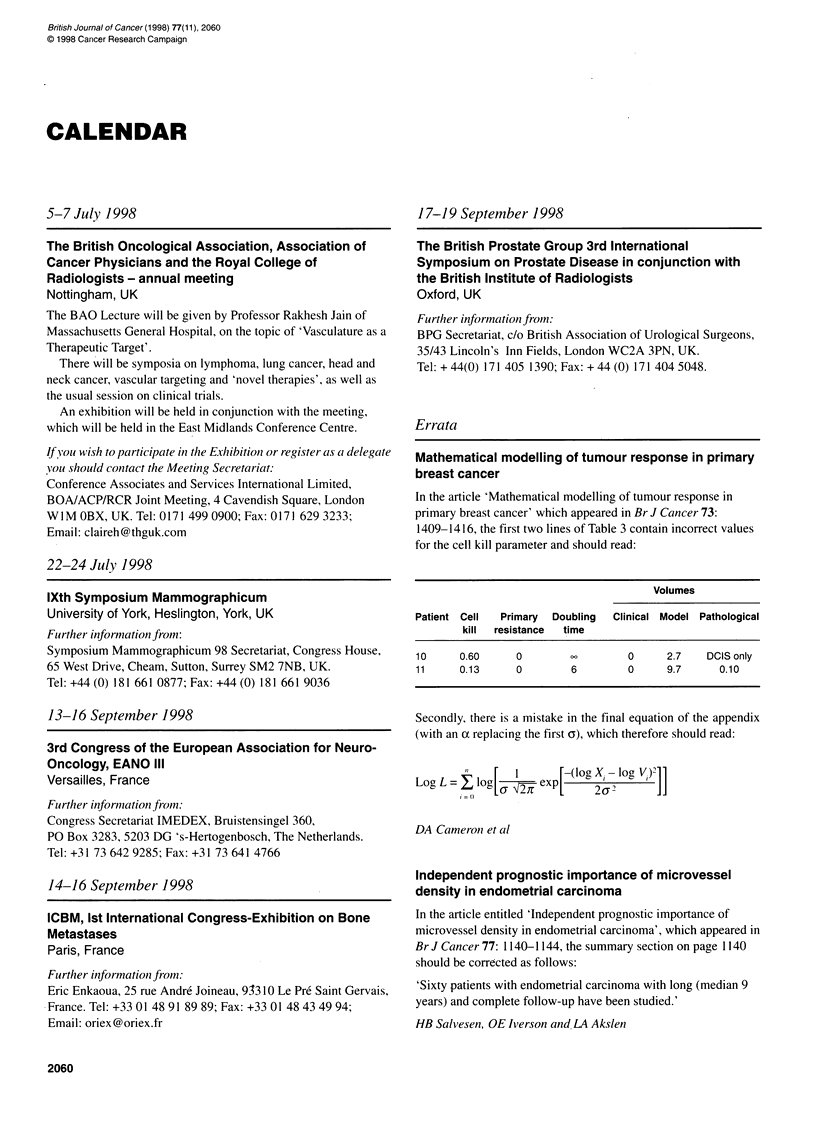# Independent prognostic importance of microvessel density in endometrial carcinoma

**Published:** 1998-06

**Authors:** 


**Independent prognostic importance of microvessel density in endometrial carcinoma**

In the article entitled 'Independent prognostic importance of microvessel density in endometrial carcinoma', which appeared in *Br J Cancer* **77**: 1140–1144, the summary section on page 1140 should be corrected as follows:

'Sixty patients with endometrial carcinoma with long (median 9 years) and complete follow-up have been studied.'

*HB Salvesen, OE Iverson and LA Akslen*